# Influence of oxygen on asexual blood cycle and susceptibility of *Plasmodium falciparum *to chloroquine: requirement of a standardized *in vitro *assay

**DOI:** 10.1186/1475-2875-6-44

**Published:** 2007-04-16

**Authors:** Sébastien Briolant, Philippe Parola, Thierry Fusaï, Marilyn Madamet-Torrentino, Eric Baret, Joël Mosnier, Jean P Delmont, Daniel Parzy, Philippe Minodier, Christophe Rogier, Bruno Pradines

**Affiliations:** 1 Unité de Recherche en Biologie et Epidémiologie Parasitaires, Institut de Médecine Tropicale du Service de Santé des Armées, Marseille, France; 2 Service de Maladies Infectieuses et Tropicales, Hôpital Nord, Marseille, France; 3 Unité de Recherche en Pharmacologie et Physiopathologie Parasitaires, Institut de Médecine Tropicale du Service de Santé des Armées, Marseille, France; 4 Service de Pédiatrie, Hôpital Nord Marseille, France

## Abstract

**Objective:**

The main objective of this study was to assess the influence of gas mixtures on *in vitro Plasmodium falciparum *growth and 50% inhibitory concentration (IC_50_) for chloroquine.

**Methods:**

The study was performed between February 2004 and December 2005. 136 *Plasmodium falciparum *isolates were used to evaluate gas mixtures effect on IC_50 _for chloroquine by isotopic microtest. The oxygen effect on asexual blood cycle of 3D7 and W2 clones was determined by thin blood smears examination and tritiated hypoxanthine uptake.

**Results:**

From 5% O_2 _to 21% O_2 _conditions, no parasiticide effect of O_2 _concentration was observed *in vitro *on the clones 3D7 and W2. A parasitostatic effect was observed during the exposure of mature trophozoïtes and schizonts at 21% O_2 _with an increase in the length of schizogony. The chloroquine IC_50 _at 10% O_2 _were significantly higher than those at 21% O_2_, means of 173.5 nM and 121.5 nM respectively (p < 0.0001). In particular of interest, among the 63 isolates that were *in vitro *resistant to chloroquine (IC_50 _> 100 nM) at 10% O_2_, 17 were sensitive to chloroquine (IC_50 _< 100 nM) at 21% O_2_.

**Conclusion:**

Based on these results, laboratories should use the same gas mixture to realize isotopic microtest. Further studies on comparison of isotopic and non-isotopic assays are needed to establish a standardized *in vitro *assay protocol to survey malaria drug resistance.

## Background

Drug resistance of *Plasmodium falciparum*, the most deadly human malaria parasite with nearly 500 millions of new clinical cases each year [1], makes malaria control more difficult [2,3]. There are basically three approaches to the assessment of the antimalarial drug susceptibility of *P. falciparum*: *in vivo *assays as defined by the World Health Organization [4], *in vitro *assays and molecular markers of resistance [5].

In a number of laboratories surveying malaria drug resistance, *in vitro *tests are performed using the uptake of a radiolabelled nucleic acid precursor [^3^H]-hypoxanthine [6] as a marker of parasite growth. Others methods can be used: the WHO schizont maturation tests by optical microscopy (Mark III) with pre-dosed plates [7], which was based on the method of Rieckmann *et al *[8] and of Wernsdorfer [9], a flow cytometric analysis of propidium iodide incorporation into parasite, which permits a stage-specific evaluation of antimalarial compounds [10], and colorimetric assays with the measurement of Histidine Rich Protein II (HRP2) by an enzyme-linked immunosorbent assay (ELISA) [11,12] and the DELI-microtest (Double-site Enzyme-linked Lactate dehydrogenase Immunosorbent assay) [13,14].

Many factors can influence the results of the chemosusceptibility tests [15]: the initial parasitaemia, the haematocrit, the time of incubation, the time point when [^3^H]-hypoxanthine is added, the use of serum substitutes and the gas mixture. Laboratories using isotopic microtest to monitor drug resistance work at different oxygen tensions: 3% O_2 _[10], 5% O_2 _[11,12], 10% O_2 _[16], in candle jars [13,14] (which corresponds to approximately 15% O_2 _[17]) and 21% O_2 _[15] (in CO_2 _incubators). WHO recommends the use of a candle jar in their *in vitro *microtests (Mark III). But all have adopted the same threshold for the resistance to antimalarial compounds under different oxygen tensions. The aim of this study was to evaluate the influence of oxygen on the asexual blood cycle and the *in vitro *chemosusceptibility of *P. falciparum *to chloroquine in order to contribute to the establishment of a standardized *in vitro *assay protocol.

## Methods

### Isolates of *P. falciparum*

Between February 2004 and December 2005, 136 *P. falciparum *isolates were obtained from patients attending the North Hospital in Marseille [18] (France). Venous blood was collected into Vacutainer ACD tubes (Becton Dickinson, Rutherford, NJ) before treatment and transported at 4°C to the laboratory in Marseilles, that is associated to the French National Malaria Reference Center. Thin blood smears were stained using a RAL kit (Réactifs RAL, Paris, France) and examined to determine parasite density. Samples with parasitaemia ranging from 0.01% to 6.2% were used to test drug sensitivity. Parasitized erythrocytes were washed three times in RPMI 1640 medium (Invitrogen, Paisley, United Kingdom). If parasitaemia exceeded 0.8%, infected erythrocytes were diluted to 0.5–0.8% with uninfected erythrocytes and resuspended in culture medium to a haematocrit of 1.5%. Susceptibility to chloroquine was determined after suspension in RPMI 1640 medium. The suspensions were supplemented with 10% human serum (AbCys, Paris, France) and buffered with 25 mM HEPES (Sigma, St. Louis, MO) and 25 mM NaHCO_3 _(Sigma). Isolates were used for 60-hr experiments at two different gas mixtures under 10% O_2_, 5% CO_2_, 85% N_2 _in a CO_2 _water jacketed incubator series II (Model 3141, Forma Scientific, Inc.) or 21% O_2_, 5% CO_2_, 74% N_2 _in a CO_2 _incubator (Model MCO-17 AIC, Sanyo).

### Parasite clones

Chloroquine sensitive 3D7 clone and chloroquine resistant W2 clone (MR4 Resource Center) were used in this study. They were maintained in continuous culture as previously described [19] at 6% haematocrit using type O+ (3D7) or type A+ (W2) human erythrocytes in the same conditions as described above at 37°C under a 5% CO_2_, 5% O_2_, 90% N_2 _gas mixture and a humidity of 95%. Culture medium was changed every day, viability and parasitaemia of cultured parasites were calculated by light microscopy analysis of blood smear stained with RAL^® ^555 (Réactifs RAL, France), (5,000 erythrocytes counted per blood smear). Blood smear pictures were performed using a digital camera (Digital Camera DXM 1200, Nikon), and analyzed with the software Lucia 4.8 (Nikon). Parasite synchronization was performed by sorbitol treatment (D-Sorbitol, ICN Biomedicals) as previously described [20]. To obtain tightly synchronized cultures, two sorbitol treatments were carried out 12 h apart. Approximately 48 h after the initial synchronization, the cultures were synchronized again to eliminate any residual schizonts. Clonality was verified every month using PCR genotyping of polymorphic genetic markers (msp1, msp2 and microsatellite loci [21,22]).

### Drug

Chloroquine diphosphate was obtained from Sigma Chemical Co. (St. Louis, Mo, U.S.A.). Two-fold serial dilutions of chloroquine were prepared in sterile, distilled water. Final concentrations ranging from 5 nM to 3200 nM were distributed in triplicate into Falcon 96-well flat-bottom plates (Becton Dickinson, Franklin Lakes, N.J.), which were dried.

### Oxygen effects on asexual blood cycle of *P. falciparum*

Synchronous cultures of 3D7 or W2 were divided in three subcultures at 2% initial parasitaemia for 3D7 and 0.5% for W2 and 3% haematocrit under three different oxygen tensions 5% O_2_, 10% O_2 _and 21% O_2 _in three different experiments with duplicates. Culture mediums were not replaced during the experiments. Blood smears were carried out at different times to evaluate parasitaemia and percentages of different stages of parasite (5,000 erythrocytes were counted per blood smear in blind by two different examiners). The same experiments (three different experiments with duplicates) were undertaken with 3D7 parasites with only ten hours exposures of rings stages, trophozoïtes stages or schizonts stages under 21% O_2_, 5% CO_2_, 74% N_2 _gas mixture and under 5% O_2_, 5% CO_2_, 90% N_2 _gas mixture in a CO_2 _water jacketed incubator series II (Model 3131, Forma Scientific, Inc.) during the rest of the cycle to assess a stage susceptibility of oxygen effect.

### Tritiated hypoxanthine uptake

200 μl of the suspension of no parasitized and parasitized erythrocytes (1% parasitaemia, 2% haematocrit) was distributed in 96-well plates (three different experiments with six measurements). Parasite growth was assessed by adding 1 μCi of [^3^H]-hypoxanthine with a specific activity of 14.1 Ci/mmol (Perkin Elmer, Meriden, NJ) to each well. Plates were incubated for different periods and at different parasites stages at 37°C under 21% O_2_, 5% CO_2_, 74% N_2 _or 10% O_2_, 5% CO_2_, 85% N_2 _gas mixture and a humidity of 95%. Duplicate wells were used to make thin blood smears to evaluate parasitaemia and parasite stages. Immediately after incubation the plates were frozen and then thawed to lyse erythrocytes. The content of each well was collected on standard filter microplates (Unifilter™ GF/B, Perkin Elmer) and washed using a cell harvester (Filtermate™ Cell Harvester, Packard, Meriden, NJ). Filter microplates were dried and 25 μl of scintillation cocktail (Microscint™ O, Perkin Elmer) was placed in each well. Radioactivity incorporated (in counts per minute, cpm) by the parasites was measured using a scintillation counter (Top Count™, Perkin Elmer).

### Drug susceptibility assays

The isotopic micro drug susceptibility test used in this study was performed as described previously [23]. The 50% inhibitory concentration (IC_50_), i.e. the drug concentration corresponding to 50% of the uptake of [^3^H]-hypoxanthine by the parasites in drug-free control wells, was determined by nonlinear regression analysis of log dose-response curves. Data were analyzed after logarithmic transformation and expressed as the geometric mean IC_50 _with 95% confidence intervals. The cut-off value for *in vitro *resistance to chloroquine is 100 nM [24].

### Statistical analysis

The different results were analyzed with the software STATA 9.0 using a Chi-square test for related samples, a Student's T test or a Wilcoxon signed-rank test (as needed) for related samples. Differences were considered statistically significant when p < 0.05.

## Results

**Figure 1:**
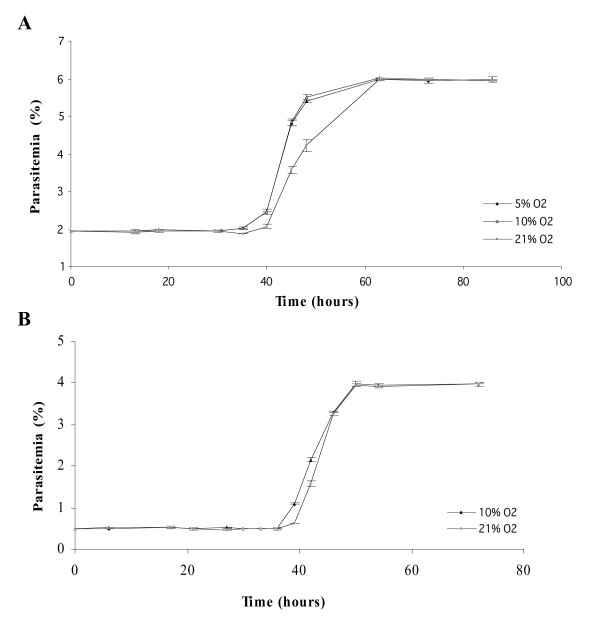
**Evolution of asexual parasitaemia of synchronous cultures of *Plasmodium falciparum *under different oxygen tensions**. Each point represents the mean ± standard deviation of three experiments. **A**: 3D7 clone. **B**: W2 clone.

**Figure 2:**
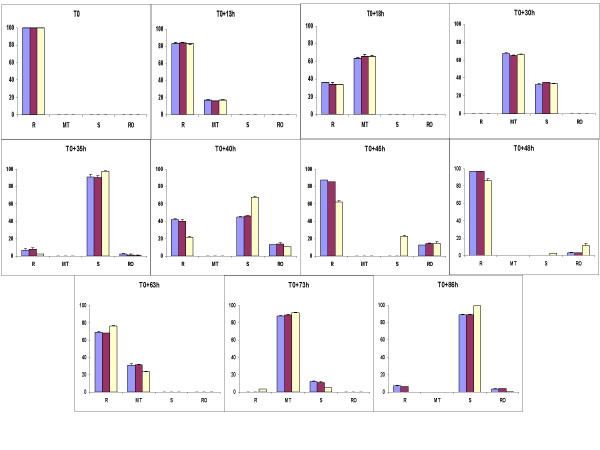
**Percentages evolution of asexual stages of synchronous culture of 3D7 *Plasmodium falciparum *clone under different oxygen tensions during 86 hours**. Each percentage represents the mean ± standard deviation of three experiments. R: Ring. MT: Mature Trophozoïte: S: Schizont. RO: Rosace. Blue histograms correspond to 5% O_2_; Purple histograms correspond to 10% O_2_. Yellow histograms correspond to 21% O_2_.

**Figure 3:**
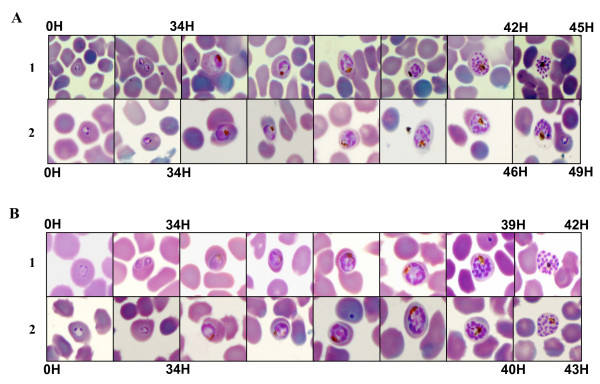
**Cell cycle evolution of asexual blood stages of *Plasmodium falciparum***. **A**: 3D7 clone. **B**: W2 clone. **1**: 5% O_2_. **2**: 21% O_2_.

**Figure 4:**
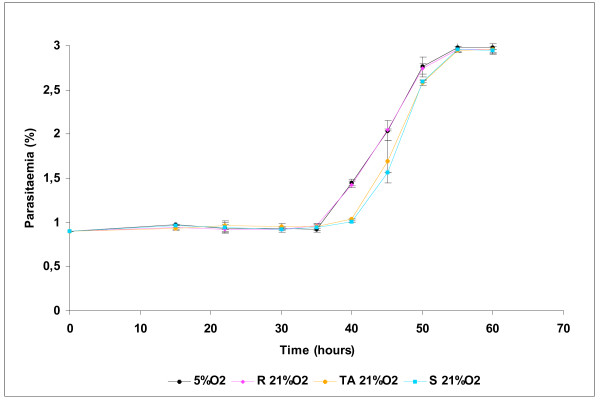
**Evolution of asexual parasitaemia of synchronous cultures of *Plasmodium falciparum *exposed to 21% O_2 _during ten hours at ring, trophozoïte or schizont stage**. Each point represents the mean ± standard deviation of three experiments.

In order to determine the effect of oxygen on asexual blood stages of *P. falciparum*, synchronous cultures of 3D7 were exposed to 5, 10 and 21% O_2 _during two cycles of parasites. As shown in Figure 1A, there were no significant difference between parasitaemia or parasite stage distribution under 5 and 10% O_2 _during all the experiment. On the contrary, from 35 h to the rest of experimental points, a significant difference between parasite stages was observed (Figure 2), for example at 40 h, there were 42% of rings and 44.9% of schizonts under 5% O_2 _and 21.7% of rings and 67.6% of schizonts under 21% O_2 _(p < 0.001, Chi-square test). After the complete reinvasion around 60 h, parasitaemia were the same in the three experimental conditions and no morphologic alteration were observed by light microscopy (Figures 3A and 3B). These results have shown that parasite exposure under 21% O_2 _had no lethal effect on parasites, but increased the length of schizogony. Moreover, only mature trophozoïtes and schizonts were susceptible to an effect of exposure to 21% O_2 _(Figure 4).

The delay was also observed with W2 clone (Figure 1B), with a briefer schizogony (8 hours for W2 and 11 hours 3D7) and a higher multiplication index for W2 (% of infected red blood cells by rings after reinvasion/% of infected red blood cells by rings at the beginning of the previous cycle), (8 for W2 and 3 for 3D7).

**Figure 5:**
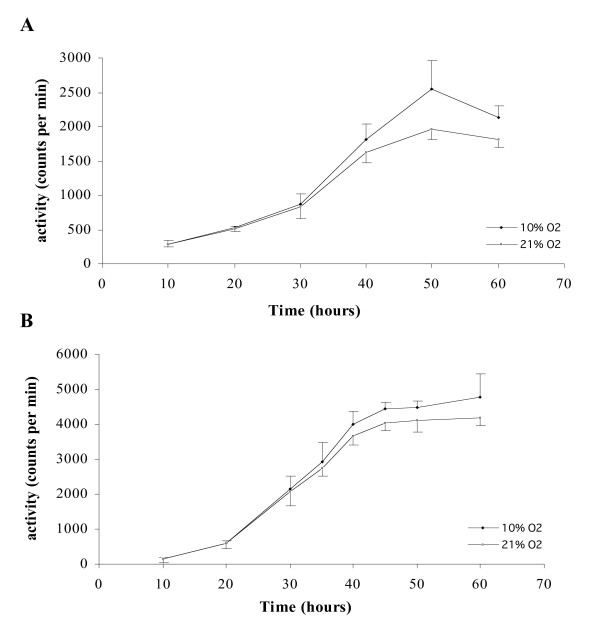
**Tritiated hypoxanthine uptake of synchronous *Plasmodium falciparum *cultures under 10% O_2 _and 21% O_2_**. Each point represents the mean ± standard deviation of three experiments. **A**: 3D7 clone. **B**: W2 clone.

No significant difference were observed in tritiated hypoxanthine uptake between 10 and 21% O_2 _with the two clones 3D7 and W2 (Figures 5A and 5B), (p = 0.087 and p = 0.76 respectively). The maximum of incorporation (around 2,500 cpm with 3D7 and 4,500 cpm with W2) was achieved at 50 hours with a steady-state until 60 hours for 3D7 and W2 in the two experimental conditions. 3D7 and W2 chloroquine IC_50 _were evaluated under 5, 10 and 21% O_2 _(three experiments). No significant difference were observed with 3D7, the IC_50 _were 15.8 nM [10.8–20.7], 14.1 nM [12.4–16.9], 17.6 nM [14.1–21.1] under 5% O_2_, 10% O_2 _and 21% O_2 _respectively. On the contrary, with W2 clone, IC_50 _under 21% O_2 _was significantly lower than IC_50 _under 5% O_2 _and 10% O_2_, 83.3 nM [15–168], 299 [228–369] and 277 [192–322] respectively (p < 0.05, Student's T test).

**Figure 6:**
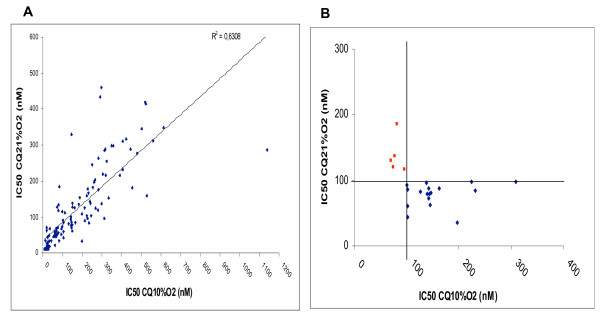
**Chemosusceptibility of *Plasmodium falciparum *isolates to chloroquine under 10% O_2 _and 21% O_2_**. **A**: chloroquine IC_50 _of 120 isolates. **B**: red plots corresponds to isolates with a switch from susceptible to chloroquine at 10% O_2 _to resistant at 21% O_2_; blue plots corresponds to isolates with a switch from resistant at 10% O_2 _to susceptible at 21% O_2_.

One hundred and thirty six *Plasmodium falciparum *isolates were used to evaluate chloroquine susceptibility at 10% O_2 _and 21% O_2_. Three tests at 10% O_2 _and 13 tests at 21% O_2 _were not interpretable, because of lack of significant difference in cpm between control-wells and higher chloroquine concentration wells. Chloroquine susceptibility of 120 isolates was finally tested at 10 and 21% O_2 _(Figure 6A). The differences between the chloroquine IC_50 _of the 120 isolates at 10% O_2 _and at 21% O_2 _were statistically significant (p < 0.0001, Wilcoxon signed-rank test), means of 173.5 nM with a standard deviation of 168.4 and 121.5 nM with a standard deviation of 106.7 respectively. The median and interquartile (Q25% & Q75%) values of the chloroquine IC_50 _at 10% O_2 _and IC_50 _at 21% O_2 _of the 120 isolates were respectively 127 nM, 30 nM, 258 nM at 10% O_2 _and 85.9 nM, 36 nM, 168 nM at 21% O_2_. In particular of interest, 52.5% of isolates had chloroquine IC_50 _> 100 nM at 10% O_2 _although 42.5% of the same isolates had chloroquine IC_50 _> 100 nM at 21% O_2_. Moreover, among the 63 isolates which had chloroquine IC_50 _> 100 nM at 10% O_2_, 17 had chloroquine IC_50 _< 100 nM at 21% O_2 _(Figure 6B) (i.e. 1/3) although five isolates only had chloroquine IC_50 _> 100 nM at 21% O_2 _among the 57 isolates which had chloroquine IC_50 _< 100 nM at 10% O_2 _(Table 1), (Chi-square test for paired samples, p < 0.025).

**Table 1:** Repartition of drug susceptibility of *Plasmodium falciparum *isolates at 10% O_2 _and 21% O_2_.

		**21% O_2_**	
		
		CQ^a ^IC_50_^b ^< 100 nM	CQ IC_50 _> 100 nM
**10% O_2_**	CQ IC_50 _< 100 nM	52	5
	CQ IC_50 _> 100 nM	17	46

^a ^CQ: chloroquine.

^b ^IC_50_: inhibitory concentration 50% (nM).

There were no significant difference between the observed control cpm at 10 (mean 12,607, standard deviation 13,425) and 21% O_2 _(mean 13,302, standard deviation 14,662).

## Discussion

The first works about oxygen effects on *P. falciparum *asynchronous cultures [17] had shown that microaerophilic environment allowed an optimal development of parasites. Their growth was impossible in strict anaerobic conditions. *P. falciparum *possesses a functional mitochondrial respiratory chain with oxygen consumption [25]. It has been shown that there is some protector effect of CO_2 _at high oxygen concentration [17] through the medium pH which stability (between 7.2 et 7.45) is required for parasite growth [26]. The analysis of the different parasites stages distribution according to the time and the oxygen concentration has allowed us to reveal a possible slow down of cellular cycle without morphologic alteration of parasites under 21% O_2_.

In the present study, the exposure to 21% O_2 _did not change parasitaemia growth rate after complete reinvasion. Previous studies [27] had shown the same absence of oxygen effect on synchronous cultures parasitaemia during four days. Here, it has been shown that the exposure of 3D7 and W2 *P. falciparum *clones to 21% O_2 _had parasitostatic effect by lengthening the schizogony. Mature stages had a particular susceptibility to high oxygen concentration. Moreover, these results justified to test drug susceptibility after a 60 hours period of incubation with [^3^H]-hypoxanthine. At the end of trophozoïtes stage, DNA replication begins and is followed by a succession of S phases during the schizogony [28]. Under 21% O_2_, the nucleic acids synthesis decreased from 30 hours for the two *P. falciparum *clones comparing to 5% O_2 _exposure. Under 5% O_2_, a low [^3^H]-hypoxanthine incorporation took place for the first twenty hours, probably corresponding to RNA synthesis in rings, followed by an increase between 20 and 30 hours in possible relation with the beginning of DNA replication at late trophozoïtes stage [29]. Between 30 and 40 hours, during schizogony, DNA synthesis increased and the peak was achieved at 50 hours [30].

In the present study, the chloroquine IC_50 _at 10% O_2 _were significantly higher than those at 21% O_2_. A previous study did not show oxygen dependent effects of chloroquine on *P. falciparum *in culture [31], but in that experiment only four strains were tested. In the present work, among the 63 isolates which had chloroquine IC_50 _> 100 nM at 10% O_2_, 17 had chloroquine IC_50 _< 100 nM at 21% O_2_. The effect of gas mixture on the results of chloroquine chemosusceptibility should led different laboratories involved in malaria resistance survey to adapt a resistance threshold for each gas mixture or to use the same conditions to perform isotopic microtests.

## Conclusion

Several factors influencing the results of the chemosusceptibility tests (the initial parasitaemia, the haematocrit, the time of incubation, the time point when [^3^H]-hypoxanthine is added, the use of serum substitutes) have already been investigated by Basco [15,32]. The present data suggest the importance of the gas mixture on isotopic microtest results for chloroquine. Further studies are needed to evaluate gas mixture impact on isolates susceptibility to other antimalarial compounds and their correlation with molecular markers of resistance and *in vivo *evaluation of drug efficacy. Other investigations about the preparation of drug solutions, the storage of pre-dosed culture plates are required before *in vitro *drug sensitivity assay can become a standardized tool for laboratories to validate the threshold for resistance in respect to the clinical responses and molecular markers.

## Conflict of interest

The author(s) declare that they have no competing interests.

## Authors' contributions

SB contributed to the design and execution of the study, data analysis and prepared the first draft of the manuscript. PP contributed to the data collect and drafting of the manuscript. TF contributed to the design of the study and drafting of the manuscript. MMF contributed to the execution of the study and writing of the manuscript. EB contributed to the execution of the study. JM contributed to the execution of the study. JPD contributed to the data collect and the intellectual content of the manuscript. DP contributed to the study design. PM contributed to the data collect and the intellectual content of the manuscript. CR contributed to the study design as well as data analysis and writing of the manuscript. BP contributed to the study design, data analysis and writing of the manuscript.

## Disclaimer

The views and opinions are those of the authors and do not purport to represent those of the French Ministry of Defense.
